# Methods for cost-efficient, whole genome sequencing surveillance for enhanced detection of outbreaks in a hospital setting

**DOI:** 10.1371/journal.pone.0355550

**Published:** 2026-09-01

**Authors:** Kady D. Waggle, Marissa Pacey Griffith, Alecia B. Rokes, Vatsala Rangachar Srinivasa, Erin M. Nawrocki, Deena Ereifej, Rose Patrick, Hunter Coyle, Shurmin Chaudhary, Nathan J. Raabe, Kathleen Shutt, Alexander J. Sundermann, Vaughn S. Cooper, Lee H. Harrison, Lora Lee Pless

**Affiliations:** 1 Microbial Genomic Epidemiology Laboratory, Center for Genomic Epidemiology, University of Pittsburgh, Pittsburgh, Pennsylvania, United States of America; 2 Division of Infectious Diseases, University of Pittsburgh School of Medicine, Pittsburgh, Pennsylvania, United States of America; 3 Department of Infectious Diseases and Microbiology, School of Public Health, University of Pittsburgh, Pittsburgh, Pennsylvania, United States of America; 4 Department of Microbiology and Molecular Genetics, University of Pittsburgh School of Medicine, Pittsburgh, Pennsylvania, United States of America; 5 Center for Evolutionary Biology and Medicine, University of Pittsburgh School of Medicine, Pittsburgh, Pennsylvania, United States of America; 6 Department of Epidemiology, School of Public Health, University of Pittsburgh, Pittsburgh, Pennsylvania, United States of America; Versiti Blood Research Institute, UNITED STATES OF AMERICA

## Abstract

**Introduction:**

Outbreaks of healthcare-associated infections (HAI) result in substantial patient morbidity and mortality; mitigation efforts by infection prevention teams have the potential to curb outbreaks and prevent transmission to additional patients. The incorporation of whole genome sequencing (WGS) surveillance of suspected high-risk pathogens often identifies outbreaks that are not detected by traditional infection prevention methods and provides evidence for transmission. Our approach to real-time WGS surveillance, the Enhanced Detection System for Healthcare-Associated Transmission (EDS-HAT), has 1) identified serious outbreaks that were otherwise undetected and 2) shown the potential to be cost saving.

**Methods:**

We describe our cost-efficient methods to perform WGS surveillance and data analysis of pathogens for institutions that are interested in expanding infection prevention surveillance. We provide an overview of the weekly workflow of EDS-HAT during two distinct phases over three years.

**Results:**

In an average week at our tertiary healthcare system, we sequenced 60 samples at a cost of less than $100 each during Phase 1, and 80 samples for less than $70 each in Phase 2, inclusive of laboratory reagents and staff salaries. The average turnaround time, from sample collection to reporting data to infection prevention, was nine days.

**Conclusions:**

Performing EDS-HAT in real-time can be both feasible and time-efficient. Providing such timely information to aid in outbreak detection could identify transmission events sooner and thus could increase patient safety.

## Introduction

Healthcare-associated infections (HAIs) are associated with substantial morbidity and mortality. HAIs also impose a significant economic burden on healthcare systems, costing hospitals an estimated $9.6 billion USD/year [1,2]. Whole genome sequencing (WGS) for HAI organisms can provide insight on the transmission dynamics in hospital settings [3–5]. Historically, determining the degree of genomic relatedness between organisms was accomplished using pulsed field gel electrophoresis (PFGE) [6]. Given its many advantages and recent decline in cost, WGS has emerged as the leading method for determining genetic relatedness between clinical isolates [7,8]. Few hospital systems perform prospective WGS routinely or for an extended time; thus, reactive sequencing is often used to confirm or refute the presence of a suspected outbreak for most hospitals. This approach can fail to detect important outbreaks for a variety of reasons, including outbreaks caused by common organisms, those not clustering on a single nursing unit, those consisting of a small number of patients, or those caused by an unsuspected and/or complex transmission route [9–11]. Furthermore, WGS can be used to infer phylogenetic relationships among organisms, detect the presence of antimicrobial resistance (AMR) genes and mobile genetic elements, and identify rare and/or novel genetic variants [10–14]. The Microbial Genomic Epidemiology Laboratory (MiGEL) at the University of Pittsburgh developed the Enhanced Detection System for Healthcare-Associated Transmission (EDS-HAT) to identify outbreaks of HAIs in real time using WGS surveillance methods in partnership with the UPMC IP&C team and the UPMC Clinical Laboratories. EDS-HAT is currently in operation at our institution and has been for over four years [10,11,14–17].

Presumed barriers for most hospital systems for implementing WGS surveillance include cost, lack of technical expertise, and inadequate infrastructure. In this paper, we describe our laboratory methods and bioinformatics workflow, alongside an operational cost estimate, with the goal of offering a comprehensive implementation framework for hospitals that seek to utilize WGS surveillance.

## Materials and methods

### Study setting

MiGEL is a non-Clinical Laboratory Improvement Amendments (CLIA) certified research laboratory located on the University of Pittsburgh main campus, in Pittsburgh PA, USA. EDS-HAT was developed and is currently implemented in real-time at MiGEL in coordination with the University of Pittsburgh, UPMC, the UPMC Clinical Laboratory Building (CLB) team, the UPMC IP&C team, and Carnegie Melon University (CMU). UPMC Presbyterian is an adult tertiary acute care hospital with 758 total beds, 134 critical care beds, and over 400 annual solid organ transplants. UPMC Presbyterian Hospital, the primary teaching hospital of the system, is located adjacent to the University of Pittsburgh’s main campus. The University of Pittsburgh Institutional Review Board provided ethics approval for EDS-HAT (Protocol: STUDY21040126; informed consent was not required). Select research staff were able to access identifiable patient information throughout the study.

The protocol described in this peer-reviewed article is published on protocols.io, https://dx.doi.org/10.17504/protocols.io.eq2lyob3pgx9/v2 and is included for printing as S1 File with this article.

### Clinical specimen collection

#### Isolate inclusion criteria.

**Fig 1:**
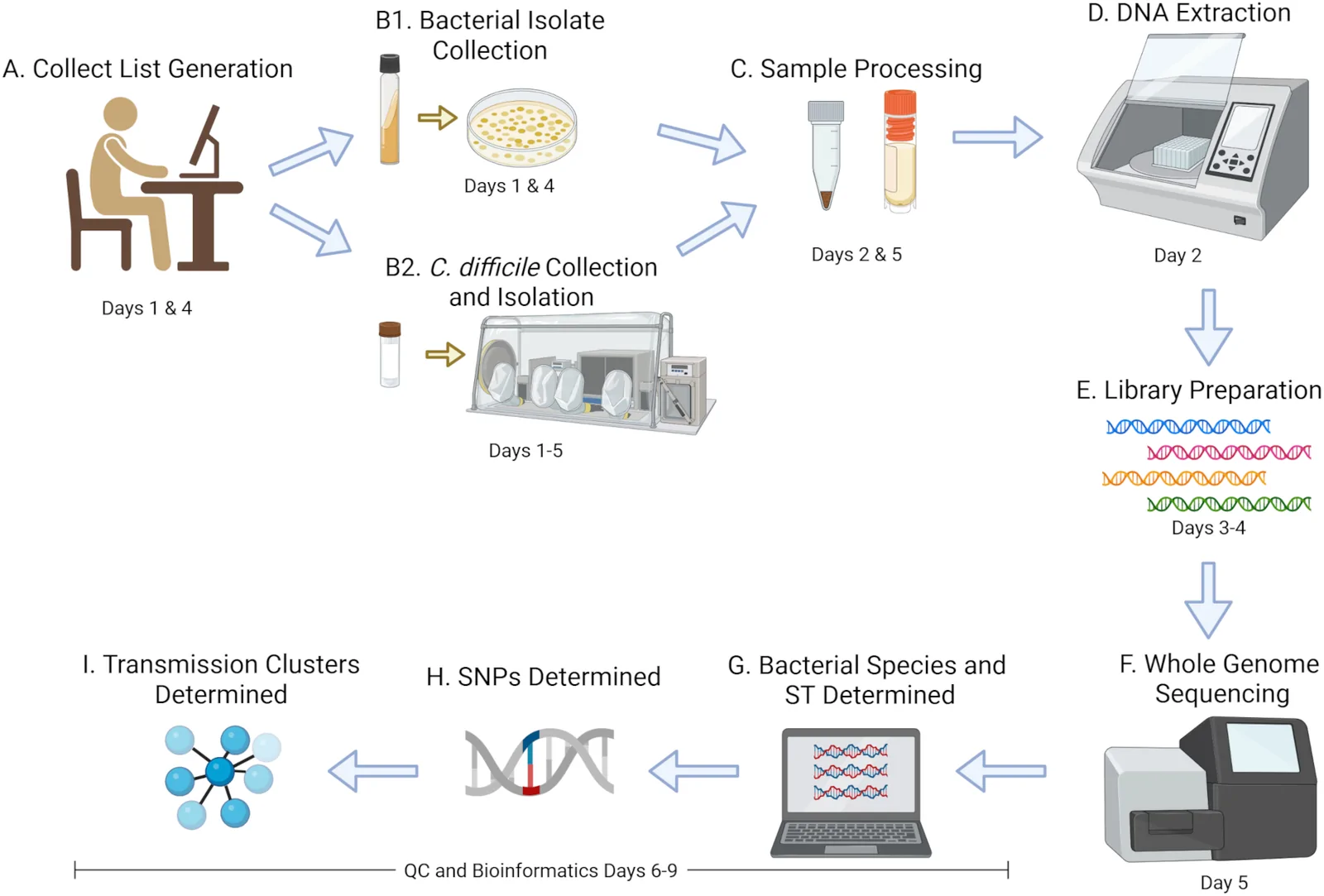
EDS-HAT real-time genomic surveillance methods. A) Collect list generation twice weekly for all EDS-HAT organisms of interest obtained from patients admitted to the hospital for ≥3 days or had a previous hospital exposure in the prior 30 days, B1) Bacterial isolate collection from the UPMC Clinical Microbiology Laboratory, B2) Clinical stool specimen collection and *C. difficile* isolation using a Coy anaerobic chamber, C) Processing samples into pellets and glycerol stocks, D) DNA extraction, E) Library preparation using Eppendorf epMotion 5075, F) WGS using MiSeq or NextSeq550 (Phase 1); NextSeq1000 or NovaSeq X (Phase 2), G) Bioinformatic analysis to determine bacterial species and sequence type (ST), H) Determination of SNPs between isolates, and I) Determination of transmission clusters. The average turnaround time from MiGEL sample collection to determination of transmission clusters was 9 days (for samples sequenced externally on the NovaSeq X Plus, the timeline was 14 days). Created with BioRender.

A list of select, high-concern bacterial pathogens from UPMC Presbyterian Hospital was generated twice weekly using Theradoc (5.4.0.HF1.102, Pittsburgh, PA; Fig 1A). Pathogens of interest included: extended-spectrum beta-lactamase-producing (ESBL) *Escherichia coli,* ESBL *Enterobacter* species, *Acinetobacter* species, *Pseudomonas* species, *Klebsiella* species, *Stenotrophomonas* species, *Serratia* species, *Burkholderia* species, *Providencia* species, *Proteus* species, *Citrobacter* species, vancomycin-resistant *Enterococcus* (VRE), methicillin-resistant *Staphylococcus aureus* (MRSA), and *Clostridioides difficile*. EDS-HAT isolate inclusion criteria required patients to have been hospitalized for ≥3 days and/or to have had a previous hospital exposure within the 30-days prior to culture [10]. In this study, we describe the samples and methods utilized to perform real-time EDS-HAT from March 1, 2022-March 1, 2025. We have described two phases, which were defined by a transition from the use of our initial sequencing instrumentation (MiSeq and NextSeq550) to more recent, higher throughput technologies (NextSeq1000 and NovaSeq X Plus). For the remainder of the manuscript, MiSeq refers to the v3 600 cycle flow cell (2 × 300 bp), NextSeq 550 refers to the mid output v2.5 300 cycle flow cell (2 × 150 bp), NextSeq 1000 refers to the P1 XLEAP-SBS 300 cycle flow cell (2 × 150 bp), and NovaSeq X Plus refers to the NovaSeq X Series 25B 300 cycle flow cell (2 × 150 bp; sequencing was performed by Azenta (South Plainfield, NJ). Details on kits and reagents are provided in Supplementary Table 1.

#### Isolate collection.

Bacterial samples were collected by MiGEL twice weekly at the CLB from pure cultures isolated from clinical specimens. (Fig 1.B1). CLB technologists subcultured all eligible gram-negative isolates from aerobic bacterial cultures to nutrient agar slants. We identified isolates of interest from the CLB, subcultured to trypticase soy agar with 5% sheep blood agar plates (BAP) (BD, Franklin Lakes, NJ), transported to MiGEL, and incubated at 37°C overnight in the presence of 5% CO_2_. The eligible gram-positive isolates from the CLB were transferred from one BAP to another and then transported and incubated at MiGEL following the same procedure. The next day, sample information was imported into the MiGEL database, and a de-identified specimen ID was generated per sample.

#### *Clostridioides difficile* collection and culture.

We collected clinical stool specimens that tested positive for *C. difficile* by culture-independent diagnostic testing [18] and performed the following protocol to isolate this organism directly from the stool. In a biosafety cabinet, stool samples were subcultured onto cycloserine-cefoxitin-mannitol-agar with taurocholate and lysozyme (CCMA-TAL) plates to select for *C. difficile* growth. Plates were transferred into a Coy anaerobic chamber (Coy Laboratory Products, Grass Lake, MI) and incubated at 37°C for 48 hours. Colonies of *C. difficile* were passaged to a second CCMA plate and incubated at 37°C in the anaerobic chamber for an additional 24–48 hours. Isolates were confirmed as *C. difficile* by testing for L-Proline aminopeptidase production using a PRO Disc test (Remel, San Diego, CA; Fig 1B2).

### Sample preparation and DNA extraction

To begin sample preparation for WGS, microcentrifuge tubes containing 750 µL phosphate buffered saline (PBS) were inoculated with a quarter-portion of a 10 µL loop of bacteria (a half-portion was used for *C. difficile*) from the BAP or CCMA plate. The tubes were centrifuged at 6,000 × *g* for 10 minutes to generate a pellet; the supernatant was removed using a P1000 pipette (Fig 1C). For samples not proceeding immediately to extraction, the pellets were stored at –20°C. Isolate stocks for long-term storage for all bacterial isolates (including *C. difficile*) were prepared by inoculating a 10 µL loop of bacteria into cryovials containing 1 mL of nutrient broth with 20% glycerol and then stored at –80°C.

The bacterial pellets were re-suspended in 500 µL PBS prior to extraction. DNA was extracted using the MagMAX DNA Multi-Sample Ultra 2.0 extraction kit on the KingFisher Apex (Thermo Fisher Scientific, Waltham, MA) per manufacturer’s instructions (Fig 1D). Briefly, this procedure isolates and purifies nucleic acids using magnetic bead-based technology. DNA was eluted in 100 µL of elution buffer and then quantified using a Qubit broad range dsDNA kit (Life Technologies, Carlsbad, CA). Samples with a concentration ≥3.5ng/µL were considered for WGS. For samples that did not meet this criterion, DNA was re-extracted.

### Library preparation

DNA libraries were prepared on an epMotion 5075t (Eppendorf, Hamburg, Germany) using a DNA Prep-(M)-Tagmentation kit (item 20060059; S1 Table; Illumina, San Diego, CA). To optimize throughput and minimize costs, we utilized a modified protocol consisting of half-volume reactions for the bead-linked transposomes (BLT/TB1) and the enhanced PCR mix (EPM) reagents (Fig 1E; S1 File), while DNA input volume and all subsequent downstream reagent volumes remain unchanged. A comprehensive list of all reagents, vendors, and item numbers is provided in S1 Table. A unique 10-mer index adapter sequence was ligated to each sample (IDT, Coralville, IA). Briefly, this protocol uses bead-linked transposomes to tagment and amplify the adapter-tagged DNA segments. To ensure uniform sequencing coverage, libraries were first combined into intermediate pools of eight by pooling 5 µL of each individual library. Each sub-pool was then quantified using a Qubit high sensitivity dsDNA kit and normalized to 4 nM with resuspension buffer (RSB), using a fragment size of 700 nucleotides, based on consistent measurements obtained from Agilent TapeStation D1000 ScreenTape. Mixing individual libraries into sub-pools was done to control against quantification variance and was consistently maintained across both study phases. These intermediate pools were then combined in equimolar concentrations into a single, final library. The distribution of the fragments in the final sequencing pool was assessed using an Agilent Tapestation D1000 ScreenTape and reagents per manufacturer’s protocol (Agilent Technologies, Santa Clara, CA).

### Whole genome sequencing

#### Phase 1 (March 2022-July 2024).

DNA libraries were sequenced weekly using an Illumina MiSeq (≤32 samples) or an Illumina NextSeq550 (33−96 samples) platform (Fig 1F). The pooled DNA library was denatured using 0.2N NaOH and spiked with 1% PhiX. The DNA library was diluted, using the average library length, to the final loading concentration of 16 pM for the MiSeq or 1.5–1.6 pM for the NextSeq550. A commercial lab was used for sequencing in rare instances where personnel were unavailable for in-house sequencing. For these occasions, DNA was extracted and sent for same-day delivery using a local medical courier service, followed by library preparation and sequencing at the commercial lab.

#### Phase 2 (July 2024-February 2025).

DNA libraries were sequenced using an Illumina NextSeq1000 (48–72 samples) or an Illumina NovaSeq X Plus (73–192 samples) platform (Fig 1F); the DNA library was spiked with 4% PhiX and diluted, using the average library length, to the final loading concentration of 850 pM for the NextSeq1000 or 300 pM for the NovaSeq X Plus. DNA libraries underwent onboard denaturation.

#### Sample count.

**Fig 2:**
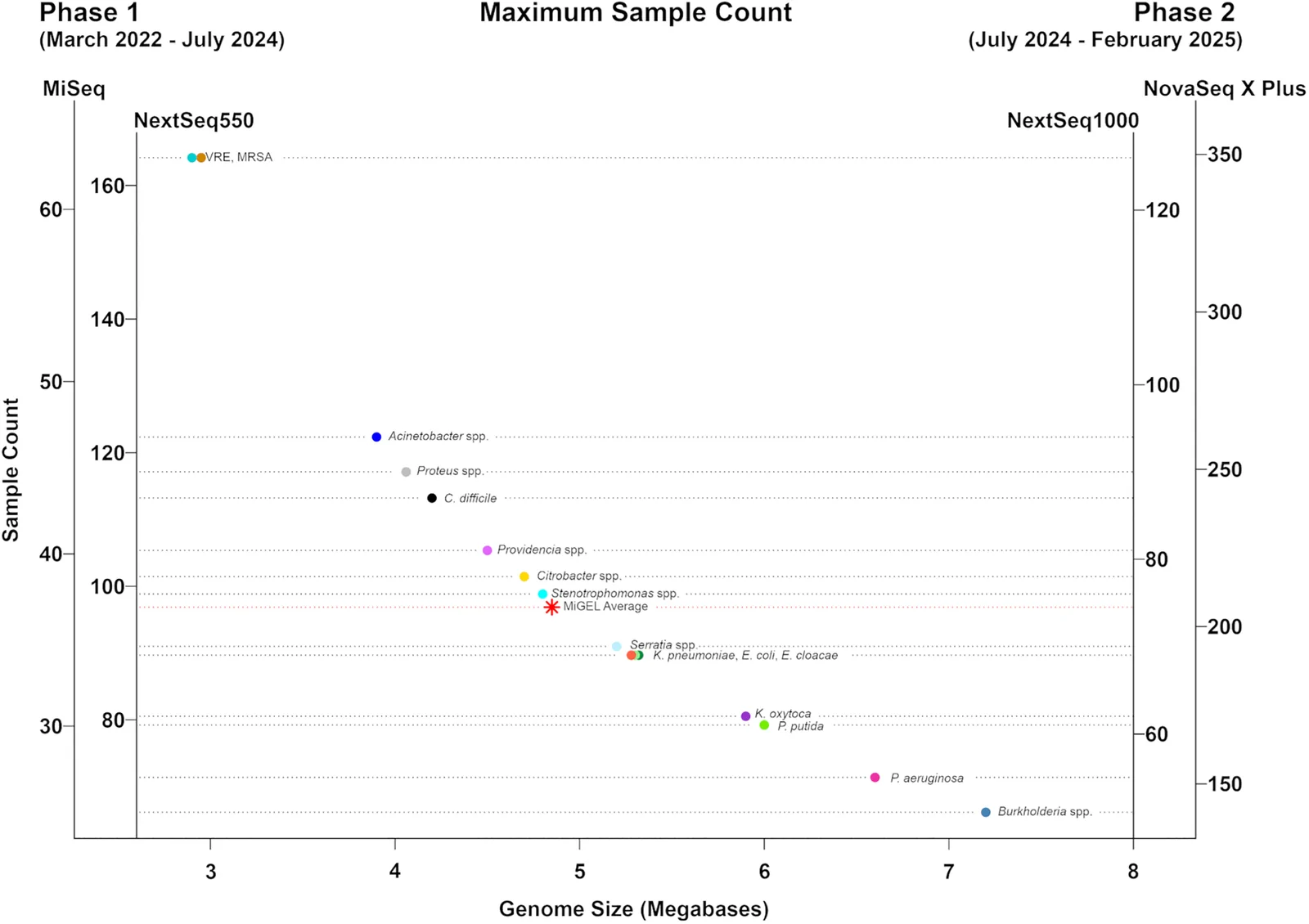
Comparative analysis of genomic multiplexing capacity between Phase 1 and Phase 2 sequencing platforms. Capacity is modeled across varying genome sizes (Mb) for Phase 1 (MiSeq and NextSeq550) and Phase 2 (NextSeq 1000 and NovaSeq X Plus service allocation) configurations. Sample counts were calculated using Illumina coverage calculator based on 80 × coverage criteria. Genome size of an average run by MiGEL is shown in comparison to individual organism sizes (red star). Note, for the NovaSeq X Plus, capacity was calculated on a standardized service allocation of approximately 350M reads, (105Gb), which represents a typical multiplexing unit provided by the vendor, rather than the total theoretical output of a full 25B flow cell.

To estimate the maximum number of samples per sequencing flow cell, we considered our weekly average genome size and approximated a minimum target of 80 × coverage per sample (Fig 2). For Phase 1, we considered a maximum of 32 or 96 samples for MiSeq or NextSeq550, respectively, and for Phase 2, a maximum of 72 or 192 samples for NextSeq1000 or NovaSeq X Plus, respectively. These reported maximum sample counts are slightly lower than the actual maximum counts to allow room for genome variability in the sequencing pool. When sequencing pools that contained a greater number of organisms with a smaller average genome size, we were able to increase the number of isolates per flow cell without compromising run quality or per organism genome coverage (Fig 2). Based on the MiGEL average genome size, and to maximize cost savings, runs containing 32–40 samples were sequenced on the MiSeq, and runs containing 40–96 samples were sequenced on the NextSeq550 platform during Phase 1. For Phase 2, runs containing 48–72 samples were sequenced on the NextSeq1000 and runs containing 72–192 samples were sequenced on the NovaSeq X Plus. During this study, 18 runs were performed on the MiSeq, 90 on the NextSeq550, 9 on the NextSeq1000, and 17 on the NovaSeq X Plus platforms.

### Bioinformatics and data analysis

#### Sequencing data quality control (QC).

We developed a publicly available Python-based bioinformatics pipeline (GitHub: https://github.com/mpgriffith/edshat-pipeline and Zenodo: https://zenodo.org/records/18557365) that was executed weekly for newly sequenced samples. Before execution of the real-time bioinformatics pipeline, the data was first downloaded from BaseSpace Sequence Hub v7.18.0 (Illumina), sample reads were demultiplexed using bcl2fastq c2.20 (Illumina) software, and results were parsed into individual directories (S1 Fig). Specific to the NovaSeq X Plus, image analysis and base calling were conducted by NovaSeq Control Software (NCS; Illumina).

#### Genome assembly and characterization.

WGS reads were assembled using Unicycler v0.5.0 and annotated using Prokka v1.14 [19]. Multilocus sequence types (STs) were assigned using PubMLST typing schemes for all organisms except *Serratia* spp. and *Providencia* spp., which do not have ST schemes (mlst v2.11) [20]. Reads were mapped using Kraken2 with the Kraken standard database to determine the most prevalent species [21].

#### Sequencing data quality control (QC).

To ensure data integrity, isolates were required to pass a standardized two-stage QC pipeline consisting of read-level and assembly-level metrics.

Read-Level QC. Raw sequencing reads were required to meet a minimum average coverage depth of 35x. Additionally, taxonomic profiling of the reads was performed using Kraken2 to confirm that the most prevalent species matched the expected organism. If a sample contained a mixture of species (defined as >10% of the reads assigned to a non-target species), we attempted to rescue the sample by using the ‘extract_kraken_reads.py’ script from the KrakenTools package [22] to isolate reads assigned strictly to the target genus, which were then re-routed through the assembly pipeline.

Assembly-Level QC. *De novo* assemblies were generated from the passing reads. Contigs measuring ≤500 bp were removed and Quast was used to determine the genome length, contig counts, and N50 [23]. CheckM was used to evaluate assembly completeness and contamination [24]. To be included in further downstream analyses, isolates passed our QC criteria if 1) the Kraken2 taxonomic assignment matched the expected organism, 2) there was at least 35 × average read depth, 3) the assembly length was within 20% of the expected genome length, 4) the assembly consisted of ≤ 350 contigs, and 5) the CheckM score had at least 95% completeness and less than 5% contamination (Fig 1G).

Data were stored on our in-house server (Dell PowerEdge T640 with 172TB hard drive space, 1.48TB RAM and two Intel® Xeon® Gold 6240R Processor CPUs).

#### Determining genetically related clusters and downstream applications.

As part of the bioinformatics pipeline, samples from the most recent sequencing batch were compared with one another and against all previously collected isolates of the same species using single nucleotide polymorphisms (SNPs) to identify instances of genetically related clusters.

Pairwise SNPs were determined using one of two methods (Fig 1H). i) Pairwise core genome SNPs (cgSNPs) were determined using Snippy v4.3.0, a reference-based method, for isolates with the same ST [25]. The genome with the highest N50 (weighted median, i.e., contigs of that length or longer contain at least 50% of the total assembly size) was chosen as the reference genome for Snippy. SNP distances were calculated from the core alignment using ‘snp-dists’ [26]. While Snippy provides high sensitivity for closely related isolates, using a reference-based mapping can generate spurious SNPs if applied across the entire species where genomes may be highly divergent. ii) SKA v1.0, a reference-free method, was used to calculate SNP distances for isolates of the same species [27]. By using SKA with all isolates of a species, we capture pairwise comparisons that might be missed due to single-locus variants of STs and species without a defined MLST schema. We selected the minimum SNP distance for each pairwise comparison quantified by Snippy or SKA to determine clusters of genetically similar isolates, minimizing the risk of missing true clusters due to limitations specific to either method. These clusters were defined using hierarchical clustering with average linkage. For all species (except *C. difficile*), the threshold was updated from ≤15 to ≤10 SNPs in May 2024 to improve the precision of cluster definitions that better aligned with epidemiological evidence [11]. *C. difficile* were evaluated using a consistent cutoff of ≤2 SNPs (Fig 1H; https://scipy.org/(10)). Phylogenetic trees were generated upon request using randomized accelerated maximum likelihood (RAxML; version 8.2.12) [28] using the general time reversible model of evolution (GTRCAT), Lewis correction for ascertainment bias, and 100 bootstrap replicates. We used AMRfinder to determine the presence of antimicrobial resistance gene sequences [29]. The electronic health records (EHR) for patients with genetically similar isolates were reviewed by trained hospital infection preventionists, who are board certified in infection control, as part of routine hospital surveillance to determine potential epidemiological links. Because sequencing data and transmission information were not deposited in the EHR, results were reported directly to the IP&C team. This allowed for timely implementation of targeted mitigation measures whenever transmission routes were identified.

#### Sequencing depth sensitivity analysis.

To assess whether increased output from the NovaSeq X Plus biased phylogenetic interpretations, we performed a downsampling validation (n = 231 isolates) across four species (*E. faecium*, *K. pneumoniae*, *P. aeruginosa*, and *S. aureus*). Original high-depth reads (average 327×) were downsampled to 52 × using the ‘seqkit sample’ command [30] to simulate lower-throughput platform output; pairwise genetic differences were compared.

### Cost analysis

A cost estimate for our optimized EDS-HAT real-time genomic surveillance methods was determined, standardized to 2025 US dollars, and included the cost of personnel, reagents, and supplies, and was analyzed comparatively for each sequencing platform used in this study (S1 Table). Equipment costs were not considered for weekly run costs and laboratories are assumed to have a basic setup, including typical molecular biology equipment and machines. Personnel costs (salary, including fringe benefits) were determined using the average pay scale of Laboratory Technician III (90% effort) and Bioinformatics Research Analyst II (50% effort) positions at the University of Pittsburgh, Pittsburgh, PA. Reagent and supply costs were determined using manufacturer pricing as of August 5, 2025.

## Results

### Weekly sequencing runs

From March 2022 to March 2025, MiGEL conducted real-time EDS-HAT, collecting and sequencing 7,850 bacterial isolates, with a weekly average sample count of *N* = 60 or 80 (Phases 1 or 2, respectively) and an average genome size of 4.87 million nucleotides. The average sequencing depth was 124.8× (SD, 75.3×) for Phase 1 and 281.0× (SD, 227.3×) for Phase 2. All samples collected were queued for sequencing, except for six isolates that were considered duplicates (same patient and species within 60 days), had insufficient DNA quality, or had observable discordant phenotypic morphology. To avoid redundant sequencing, isolates were excluded if the same species had already been sequenced from that patient within the preceding 60 days. The mean overall observed WGS failure rate was 2.62% (standard deviation, SD = 0.005; 206 failures/7,850 total). The most common reasons for failure included low genome depth (<35 × average nucleotide depth; 1.06%; 83 failures/7,850 total), mixed species or an unexpected species designation as determined by the clinical lab (0.90%; 71 failures/7,850 total), or poor assembly (>350 contigs) (2.19%; 172 failures/7,850 total). The most commonly sequenced organism was *Pseudomonas aeruginosa* (*N* = 2,115) and the least sequenced was *Burkholderia* spp. (*N* = 36; Table 1). The average turnaround time to complete the EDS-HAT workflow from MiGEL sample collection to bioinformatic analysis was approximately 9 days (for samples sequenced externally on the NovaSeq X Plus, the timeline was 14 days), with an average instrument run time of ~25 and ~16 hours for NextSeq550 and NextSeq1000, respectively (S2 Table). A representative example of the standardized genomic surveillance report format used to convey these cluster assignments and pairwise SNP differences to the IP&C team is provided in S2 Fig.

**Table 1:** EDS-HAT organisms sequenced by MiGEL (March 1, 2022 to February 28, 2025).

Organism	Genome Size (Mb)	Count Sequenced by MiGEL	Failures		
			Count	% by Species	% of Total
*Acinetobacter* species	3.9	174	8	4.60	0.10
*Burkholderia* species	7.2	36	6	16.67	0.08
*Clostridioides difficile*	4.2	473	34	7.19	0.43
*Citrobacter* species	4.7	156	5	3.21	0.06
*Enterobacter cloacae*	5.3	138	2	1.45	0.03
*Escherichia coli*	5.3	435	8	1.84	0.10
*Klebsiella oxytoca*	5.9	74	0	0	0
*Klebsiella pneumoniae*	5.3	554	7	1.26	0.09
MRSA	2.9	1,079	25	2.32	0.32
*Proteus* species	4.06	938	17	1.81	0.22
*Providencia* species	4.5	107	2	1.87	0.03
*Pseudomonas aeruginosa*	6.7	2,115	42	1.99	0.54
*Pseudomonas* species	6.6	87	9	10.34	0.11
*Serratia* species	5.2	534	14	2.62	0.18
*Stenotrophomonas* species	4.8	357	20	5.60	0.25
VRE	2.9	593	7	1.18	0.09
**GRAND TOTAL**		**7,850**	**206**	**–**	**2.62**

Bacterial genome sizes and the total isolates processed for sequencing are tabulated by organism. An additional six isolates were excluded prior to sequencing due to: i) duplicate identification (same patient and species within 60 days), ii) insufficient DNA quality, or iii) discordant phenotypic morphology. Results for isolates failing post-sequencing quality control are tabulated (206 failures/7,850 total). Mb, megabase.

### Data output

For an average Phase 1 run of 32 or 65 samples, we observed an average output of 12 Gb and 40 Gb of data and an average of 77 million and 257 million reads on the on the MiSeq or NextSeq550, respectively (S2 Table). For an average Phase 2 run of 70 or 84 samples, we observed an average output of 38 Gb and 117 Gb of data and an average of 244 million and 392 million reads on the NextSeq 1000 or NovaSeq X Plus, respectively (S2 Table).

Since the runs completed on the NovaSeq X Plus produced a greater number of reads compared to the other platforms, we performed a downsampling experiment to evaluate potential bias in transmission inference. The original NovaSeq X Plus data were downsampled from the average of 327x to ~52x for 231 samples across four species (*E. faecium*, *K. pneumoniae*, *P. aeruginosa*, and *S. aureus*) to assess phylogenetic stability. Between the original and downsampled datasets, pairwise SNP differences between 0–2,000 showed a correlation *R*^*2*^ = 0.98 (mean absolute error, *MAE* = 11.2); for the 0–20 SNP difference range, critical for identifying transmission clusters, *R*^*2*^ = 0.95 (*MAE* = 0.73; S3 Fig). We observed two minor discrepancies near the 10 SNP threshold, where samples shifted to 12 and 14 SNPs in the downsampled data. This could be due to coverage gaps at lower depths leading to missed SNP calls. In practice, we mitigate these borderline variances (MAE = 0.73) by requiring concordant epidemiological evidence.

### Bioinformatic analyses run time

We performed bioinformatics analyses using 40 CPU cores; the time required to completion was approximately 44 h (*N* = 72 samples). The QC pipeline took 2.2 h, on average, per sample. An average run of 72 samples using 40 cores took 32 h. Cluster analyses took 12 h, which included a per sample comparison to other samples of the same species in our EDS-HAT database (Table 1). The times required for each step or program are detailed in S3 Table.

### Cost analysis

The cost to run real-time EDS-HAT weekly was categorized into sample processing, DNA extraction and quantification, library preparation, flow cell cost, and personnel (Table 2). The programs used for the bioinformatic analyses were freely downloadable, therefore our only upfront cost was an investment in a server.

**Table 2:** Weekly cost estimates per sample (March 2022–February 2025).

	Phase 1: March 2022 – July 2024					Phase 2: July 2024 – February 2025				
Method	MiSeq Cost	NextSeq550 Cost			Commercial Lab 1 Sequencing Cost^#^	NextSeq 1000 Cost	Commercial Lab 2 (NovaSeq X Plus) Cost			Commercial Lab 1 Sequencing Cost^#^
	32 samples	48 samples	60* samples	96 samples	≤32 samples	72 samples	80* samples	96 samples	192 samples	≤32 samples
**Sample Processing**	$2.25	$2.25	$2.25	$2.25	$2.25	$2.25	$2.25	$2.25	$2.25	$2.25
**DNA Extraction & Quantification**	$9.35	$8.66	$8.39	$7.98	$9.35	$8.21	$8.11	$7.98	$7.98	$9.35
**Library Preparation**	$22.54	$21.78	$21.47	$21.01	$75	$21.26	$21.16	$21.01	$21.01	$65
**Sequencing Kit** **(Flow Cell)**	$58.47	$43.13	$34.50	$21.56		$13.89	$14.65	$12.21	$6.10	
**LABORATORY COST PER SAMPLE**	$92.61	$75.82	$66.61	$52.80	$86.60	$45.61	$46.17	$43.45	$37.34	$76.60
**Personnel**										
laboratory technician, 90% effort ($970/week)	$30.31	$20.21	$16.17	$10.10	$30.31	$13.47	$12.13	$10.10	$5.05	$30.31
bioinformatician, 50% effort ($524/week)	$16.38	$10.92	$8.73	$5.46	$16.38	$7.28	$6.55	$5.46	$2.73	$16.38
**TOTAL COST PER SAMPLE**	**$139.30**	**$106.95**	**$91.51**	**$68.36**	**$133.29**	**$66.36**	**$64.85**	**$59.01**	**$45.12**	**$123.29**

#used for comparison only, not included in calculations for averages; *average sample count per phase.

#### Phase 1.

The lowest laboratory cost per sample ($53) was achieved when the maximum number of samples (*N* = 96) was sequenced using the NextSeq550 platform. Costs ranged from $53-$93 per sample, depending on the platform and sample counts.

#### Phase 2.

The lowest laboratory cost per sample ($40) was achieved if 192 samples were sequenced using the NovaSeqX Plus platform. Costs ranged from $37-$46 per sample.

**Fig 3:**
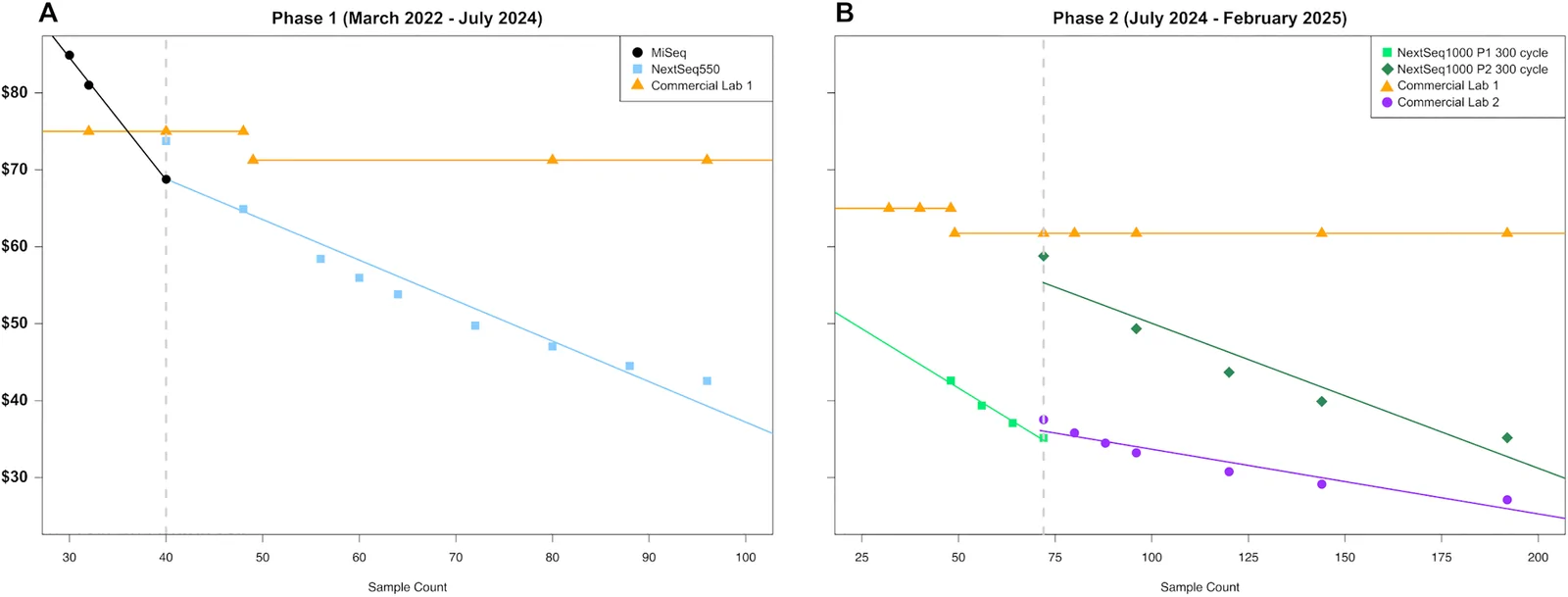
Whole genome sequencing cost per sample comparisons. A) MiSeq, NextSeq550, and commercial laboratory 1**.** Throughput cutoff between platforms is shown at *N* = 40 samples (gray dashed line). B) NextSeq1000 P1 300 cycle and P2 300 cycle flow cells, Commercial laboratory 1 and NovaSeq X Plus. Throughput cutoff between platforms is shown as *N* = 72 samples (gray dashed line). Commercial lab 1 used by MiGEL offers a discount of 5% for orders ≥ 48 samples (as of September 2025). Data points represent instances of cost by sample count and lines of best fit are shown for each sequencing method. Note: NextSeq 1000 P2 (300 cycle) flow cell is included for comparative cost-modeling purposes only.

We determined a sample count cutoff, per phase, to decide which sequencer to use for each run. For Phases 1 or 2, the sample count cutoffs were 40 or 72 samples, respectively (Fig 3). There was an inverse relationship, in both phases, between the number of samples sequenced and flow cell cost, as per sample costs significantly decreased when a greater number of samples were multiplexed on the appropriate flow cell.

The estimated weekly cost of personnel, based on the pre-tax salaries for one lab technician and one bioinformatician considering percent efforts and fringe benefits, totaled $1,494 ($970 and $524, respectively). When these costs were tabulated, the Phase 1 cost to run EDS-HAT on an average week of sequencing 60 samples totaled $5,491 ($285,511 per year), while Phase 2 costs totaled $5,188 for an average week of sequencing 80 samples ($269,755 per year).

## Discussion

In this study, we detailed an efficient laboratory workflow, our approach for bioinformatics analyses, and estimated the cost associated with implementing real-time WGS surveillance for pathogenic bacteria in a hospital system. EDS-HAT began in 2016 as a retrospective study [10] and, once we demonstrated the superiority of the system over traditional approaches, transitioned in November 2021 to a real-time workflow, subsequent bioinformatic analyses, and reporting of results to the hospital IP&C team [11]. MiGEL has been performing prospective WGS surveillance for multiple organisms in real time for the UPMC hospital system for over four years. By doing so, our hospital system has dramatically changed the way outbreaks are being detected and interrupted [10,11,31].

We provide details about our methods for a three-year timeframe, after our initial optimization period, beginning in March 2022. We divided this timeframe into two distinct phases, represented by our transition from smaller instrumentation (MiSeq and NextSeq550) in Phase 1 before expanding our throughput and upgrading to the NextSeq1000 and NovaSeq X Plus in Phase 2. We determined that the per sample cost for WGS ranged from $53 to $93 (average week: $67; Phase 1), and $37 to $46 (average week: $46; Phase 2). Furthermore, with the addition of staff salaries, the weekly cost for an average week of real-time sequencing and analyses was $5,491 (*N* = 60 samples; Phase 1) that decreased to $5,188 (*N* = 80 samples; Phase 2). Our lower cost was achieved, in part, by increasing sample counts per flow cell while utilizing the appropriate instrument and by using half-volumes of some reagents during library preparation. The transition to larger sequencing instruments also provided the use of more efficient sequencing chemistry technologies.

During Phase 1, we showed an estimated cost savings of $79,227–$285,160 per year by implementing a real-time WGS surveillance system, which was based on an average cost of $71 for sample preparation and sequencing (adjusted to 2025 USD) [31]. Since then, we have made further improvements in Phase 2 (average costs were $48/sample on the NextSeq1000 [SD, $2.28], and $45/sample on the NovaSeq X Plus [SD, $3.50]). 3/20/2014 7:02:00 PM

A major concern of hospitals with implementing programs like EDS-HAT is high cost. While sequencing was expensive years ago, the cost has significantly decreased over time [32]. Furthermore, the cost of treating preventable hospital infections is high, and, in fact, EDS-HAT has been shown to be cost saving [31,33]. Our cost efficiency at the laboratory level provides the baseline to implicate downstream economic benefits. In addition, the associated laboratory and bioinformatics methods have become more streamlined, automatable, and efficient. With our quick turnaround time from the day the sample is collected by MiGEL, we provide evidence of outbreaks that helps to guide our hospital IP&C team to implement interventions. Taken together, this approach can identify important, otherwise-undetected outbreaks in a cost-effective manner, suggesting that WGS surveillance should eventually become standard practice in hospitals. More importantly, stopping transmission events quickly at the first sign of an outbreak cluster has the potential to reduce further spread of the infection and thus, reduce patient morbidity and mortality.

To accompany our methods, we computed Phase 2 cost per sample, including staff salaries, as $65 on an average week (range, $45-$66). This cost is specific for our hospital and could be different at other locations as summarized by Price and colleagues, who determined that the cost to perform WGS varies by country and city [34]. For example, the prior cost-per-sample study, when converted to 2025 USD, showed the per sample sequencing cost ranged from approximately $59-$387 for the US and Italy, respectively. In this study, we determined our current average per sample cost (without considering staff salaries, for comparison) was $44. The primary factors in determining this cost estimate were sample counts per run and average organism genome size. Fig 2 provides a capacity guide for four Illumina platforms, using an 80 × target depth to calculate the maximum multiplexing limits based on organism and genome size. In addition, Fig 3 shows that a sample count of 40 or 72 are appropriate cutoffs to decide which sequencing platform to use for Phase 1 and Phase 2 platforms, respectively, while maintaining sufficient genome coverage. Moreover, we included a theoretical cost projection for the NextSeq 1000 P2 flow cell in Fig 3B to demonstrate how higher throughput impacts cost efficiency for other laboratory settings. Generally, we find sequencing more samples at once reduced the cost of sequencing per sample, with the exception of utilizing specific commercial labs. While one commercial lab offered a discounted price once the sample count reached 48, we found the fixed price was overall more costly than performing in-house sequencing (Fig 3). Furthermore, we found sequencing costs decreased over time. MiGEL estimated a $72 average per sample cost in 2021, which dropped to $65 in 2023 and is now further reduced to $37 in 2025. Transitioning to higher throughput sequencing platforms (NovaSeq X Plus) did not show bias for our phylogenetic interpretations.

We note limitations with this study. First, regarding local cost variations, some of the reagent and supply costs presented in this manuscript represent discounted pricing negotiated by our university; other institutions could experience different pricing structures. Furthermore, these estimates reflect specific material and salary costs in Pittsburgh, PA, which must be scaled appropriately based on geographic location and institutional overhead prior to external implementation. Second, our laboratory methods and sequencing schedules are optimized around an average weekly sample volume for our hospital system; fluctuations in weekly throughput directly impact both the turnaround time and batch-associated costs. Additionally, while MiGEL uses robotic instruments for nucleic acid extractions and library preparation to streamline turnaround times and reduce manual pipetting errors, institutions relying on manual processing will need to accommodate the laboratory methods accordingly; however, we do not think this represents a significant detriment to the process. Third, we only considered Illumina-based technology for this study. Other emerging short-read sequencing technologies or long-read sequencing were not assessed. While sequencing technologies can change rapidly, our framework is designed to be adaptable, as evidenced by our transition across historical operational phases to consistently lower per-sample costs. Fourth, due to budgetary constraints, we did not run a Tapestation analysis on the initial library pool to determine fragment size prior to the first dilution. Assuming an average fragment size of 700 bp can alter cluster density and thus affect the efficiency of the sequencing run, although we have not noticed significant negative effects to this regard. Finally, these models demonstrate a cost-effective strategy for an academic, tertiary care hospital and may not seamlessly translate to smaller hospitals. Moreover, EDS-HAT would run optimally if WGS was performed on all clinical isolates, including those from colonization [35]; however, specific species and clinical cultures were prioritized due to budgetary limitations. Finally, we achieved our lowest cost outsourcing to a NovaSeq X Plus platform. Budgetary considerations restricted our ability to purchase this machine; thus, the instrument’s select WGS metrics are unknown and therefore not included in this analysis.

In conclusion, we have shown the methods and our costs to implement a real-time WGS surveillance program. Healthcare institutions wishing to do the same could potentially identify outbreaks that would otherwise be missed. Further adoption of this approach has the potential to significantly enhance patient safety.

### Impact statement

Whole genome sequencing (WGS) surveillance to confirm or refute suspected outbreaks of potential healthcare-associated infections (HAI) is a highly effective approach for outbreak detection. Since November 2021, we have conducted WGS surveillance in real-time through a program called the Enhanced Detection System for Hospital-Associated Transmission (EDS-HAT), to assist our hospital infection prevention and control (IP&C) team to identify and stop outbreaks. Our laboratory has successfully implemented real-time WGS surveillance of multiple pathogens in the hospital setting continuously for over four years. Our weekly workflow included identifying HAI pathogens and performing WGS, followed by bioinformatic analyses that included species confirmation, determination of sequence type, and genetic relatedness comparisons. Based on this information, transmission clusters were identified, and the electronic health record was reviewed by the hospital IP&C team, to determine probable transmission routes. Finally, IP&C implemented appropriate interventions to mitigate the spread of infection. The focus of this manuscript is to provide the details of our laboratory and analytical methods, along with the cost associated with laboratory materials and staff salary, for successful implementation of real-time WGS surveillance.

### Data summary

The whole genome sequencing data generated in this study are deposited in the United States National Institutes of Health, National Library of Medicine (https://www.ncbi.nlm.nih.gov/bioproject), and are publicly available under BioProject accession PRJNA475751. All supporting data and protocols are provided within the article or through supplementary data files.

## Supporting information

S1 Table Vendor information and the reagents used to perform whole genome sequencing of bacterial isolates. (DOCX)

S2 Table Phase 1 (2a). Sequencing metrics per platform using the average number of samples (*N* = 60) sequenced by MiGEL. Phase 2 (2b). Sequencing metrics per platform using the average number of samples (*N* = 80) sequenced by MiGEL.
(DOCX)

S3 Table Bioinformatics pipeline performance metrics. (DOCX)

S1 Fig EDS-HAT bioinformatics pipeline. (TIF)

S2 Fig Representative operational genomic surveillance cluster data output. Example output provided to the IP&C team (**A**) Isolate information and cluster assignments table detailing multi-locus sequence types (ST), genomic cluster IDs, SNP distances, and binary indicator for whether clustered isolate is a newly sequenced sample. (**B**) Pairwise SNP distance matrix showing genetic distances between isolates. (**C**) Phylogenetic tree visually displays the genetic relationships and clustering hierarchy of the sequenced isolates.
(TIF)

S3 Fig Downsampling validation of high throughput sequencing output. Correlation of pairwise SNP differences between high-depth NovaSeq X Plus reads (mean 327×) downsampled (~52×) for 231 samples of four species. Inset shows SNP difference ranged from 0–20, critical for transmission inference (*R*^*2*^ = 0.95, mean absolute error = 0.73). Comparisons were performed within respective sequence types (ST) for each species. Data points represent pairwise comparisons, color coded per species, and the identity line (y = x) is shown as a gray dashed line.
(TIF)

S1 File Stepwise protocol, also available on protocols.io. https://dx.doi.org/10.17504/protocols.io.eq2lyob3pgx9/v1.
(PDF)
