# Supplementary material for: Methods for cost-efficient, whole genome sequencing surveillance for enhanced detection of outbreaks in a hospital setting
Source: PLoS One. 2026 Sep 1;21(9):e0355550. doi: 10.1371/journal.pone.0355550 (PMC13533342; doi:10.1371/journal.pone.0355550)
Supplement: Supplementary file 1 [file pone.0355550.s007.pdf]

Jul 28, 2026 Version 2

Methods for Cost-Efficient Whole Genome Sequencing Surveillance for Enhanced Detection of Outbreaks in a Hospital Setting V.2

DOI
https://dx.doi.org/10.17504/protocols.io.eq2lyob3pgx9/v2

Kady Waggle[1,2,3], Marissa Pacey Griffith[1,2], Alecia Rokes[4,5], Vatsala Rangachar Srinivasa[1,2,6], Erin Nawrocki[4,5], Deena Ereifej[1,2,6], Rose Patrick[1,2], Hunter Coyle[1,2], Shurmin Chaudhary[1,2], Nathan Raabe[1,2,6], Kathleen Shutt[1,2], Alexander Sundermann[1,2], Vaughn Cooper[4,5], Lee Harrison[1,2,6], Lora Pless[1,2]

[1]Microbial Genomic Epidemiology Laboratory, Center for Genomic Epidemiology, University of Pittsburgh, 3507 Victoria Street, Pittsburgh, Pennsylvania, USA;
[2]Division of Infectious Diseases, University of Pittsburgh School of Medicine, 3550 Terrace Street, 818 Scaife Hall, Pittsburgh, Pennsylvania, USA;
[3]Department of Infectious Diseases and Microbiology, School of Public Health, University of Pittsburgh, 130 De Soto Street, Pittsburgh, Pennsylvania, USA.;
[4]Department of Microbiology and Molecular Genetics, University of Pittsburgh School of Medicine, Pittsburgh, PA, USA;
[5]Center for Evolutionary Biology and Medicine, University of Pittsburgh School of Medicine, Pittsburgh, PA, USA;
[6]Department of Epidemiology, School of Public Health, University of Pittsburgh, 130 De Soto Street, Pittsburgh, Pennsylvania, USA

EDS-HAT

Kady Waggle

Create & collaborate more with a free account

Edit and publish protocols, collaborate in communities, share insights through comments, and track progress with run records.

Create free account

OPEN ACCESS

DOI: https://dx.doi.org/10.17504/protocols.io.eq2lyob3pgx9/v2

Protocol Citation:
Kady Waggle, Marissa Pacey Griffith, Alecia Rokes, Vatsala Rangachar Srinivasa, Erin Nawrocki, Deena Ereifej, Rose Patrick, Hunter Coyle, Shurmin Chaudhary, Nathan Raabe, Kathleen Shutt, Alexander Sundermann, Vaughn Cooper, Lee Harrison, Lora Pless 2026. Methods for Cost-Efficient Whole Genome Sequencing Surveillance for Enhanced Detection of Outbreaks in a Hospital Setting. **protocols.io**
https://dx.doi.org/10.17504/protocols.io.eq2lyob3pgx9/v2
Version created by
Kady Waggle

License: This is an open access protocol distributed under the terms of the **Creative Commons Attribution License**, which permits unrestricted use, distribution, and reproduction in any medium, provided the original author and source are credited

Protocol status: Working
We use this protocol and it's working

Created: July 28, 2026

Last Modified: July 28, 2026

Protocol Integer ID: 321609

Keywords: efficient whole genome sequencing surveillance, efficient whole genome sequencing surveillance for enhanced detection, outbreaks of healthcare, infection prevention surveillance, data analysis of pathogen, data to infection prevention, outbreak detection, mitigation efforts by infection prevention team, whole genome sequencing, infection prevention team, incorporation of whole genome sequencing, risk pathogen, serious outbreak, enhanced detection system for healthcare, outbreak, pathogen, infection prevention, wgs surveillance, associated infection, surveillance, traditional infection prevention method, time wgs surveillance, reporting data, tertiary healthcare system, healthcare, hospital setting introduction

Funders Acknowledgements:
National Institutes of Health
Grant ID: R01AI127472
National Institutes of Health
Grant ID: R21AI109459
National Institutes of Health
Grant ID: R21AI178369

Abstract

Introduction. Outbreaks of healthcare-associated infections (HAI) result in substantial patient morbidity and mortality; mitigation efforts by infection prevention teams have the potential to curb outbreaks and prevent transmission to additional patients. The incorporation of whole genome sequencing (WGS) surveillance of suspected high-risk pathogens often identifies outbreaks that are not detected by traditional infection prevention methods and provides evidence for transmission. Our approach to real-time WGS surveillance, the Enhanced Detection System for Healthcare-Associated Transmission (EDS-HAT), has 1) identified serious outbreaks that were otherwise undetected and 2) shown the potential to be cost saving.

Methods. We describe our cost-efficient methods to perform WGS surveillance and data analysis of pathogens for institutions that are interested to expand infection prevention surveillance. We provide an overview of the weekly workflow of EDS-HAT during two distinct phases over three years.

Results. In an average week at our tertiary healthcare system, we sequenced 60 samples at a cost of less than $100 each during Phase 1, and 80 samples for less than $70 each in Phase 2, inclusive of laboratory reagents and staff salaries. The average turnaround time, from sample collection to reporting data to infection prevention, was ten days.

Conclusions. Performing EDS-HAT in real-time can be both feasible and time-efficient. Providing such timely information to aid in outbreak detection could identify transmission events sooner and thus could increase patient safety.

General Notes:

1 All steps performed at room temperature, unless otherwise stated

Clinical Specimen Collection

2 **Collection List Generation**

Clinical Specimen Collection

2.1 A list of select high-concern pathogens that meet our collection criteria is generated twice per week (Theradoc, v5.4.0.HF1.102).

3 **Collecting bacterial isolates from the clinical laboratory**

3.1 Locate nutrient agar slants or blood agar plates (BAP) in the clinical laboratory that correspond to samples on the collection list.

3.2 Use 1µL loop to transfer bacteria from nutrient agar slant or BAP to a fresh BAP.

3.3 Incubate at 37°C with 5% CO_2 overnight.

3.4 Observe plates for growth and correct morphologies for expected organism.

3.5 De-identify and log isolates into database.

3.6 Reserve plates for Step 5.1 (Sample Preparation and DNA Extraction).

4 ***Clostridioides difficile* collection and culture**

4.1 Locate clinical stool specimens that correspond to samples on the collection list.

4.2 In a biosafety cabinet, use 10µL loop to streak stool onto cycloserine-cefoxitin-mannitol agar with taurocholate and lysozume (CCMA-TAL) plates.

4.3 Use 1mL disposable transfer pipette to transfer stool to 1mL cryovial for long-term storage at -80°C.
NOTE: if stool consistency is too viscous to pipette, add PBS in 1mL increments until desired consistency is reached.

4.4 Transfer inoculated CCMA-TAL plates into Coy anaerobic chamber via airlock.

4.5 Bring additional CCMA-TAL plates into Coy anaerobic chamber to pre-reduce (n=2 plates per isolate).

4.6 Incubate within anaerobic chamber at 37°C for 48h.

4.7 Transfer one colony to two new CCMA-TAL plates (CCMA-TAL_plate1 and CCMA-TAL_plate2) using a 1µL flex loop and incubate within Coy anaerobic chamber at 37°C for 24h-48h.

4.8 **Perform Prodisc procedure on CCMA-TAL_plate1**

4.9 Place one Prodisc on lid of plate using sterile forceps.

4.10 Use 1mL disposable transfer pipette to place one drop of sterile H_2O directly onto disc.

4.11 Collect bacterial colonies using the wooden end of a sterile swab.

4.12 Inoculate Prodisc with bacterial colonies by dabbing and rolling wooden end of swab on top of disc.

4.13 Incubate 2 min at room temperature.

4.14 Add 1 drop of cinnamaldehyde reagent to Prodisc (approx 20µL).

4.15 Incubate 1 min at room temperature.

4.16 Observe color change (Pink = *C. diff* positive; No change (white/yellow) = *C. diff* negative).

4.17 Plates that show color change (i.e. *C. diff* positive) are selected for the next step (Sample Preparation and DNA Extraction).

4.18 If Prodisc result is positive in step 4.17, Use a 10µL loop to gather colonies from CCMA-TAL_plate2 and inoculate in a cryovial containing 1mL nutrient broth with 20% glycerol for long-term storage at -80°C.

Sample Preparation and DNA Extraction

5 **Sample Preparation**

5.1 Starting material: plates from steps 3.6 and 4.18

5.2 Use a 10µL loop to obtain a quarter-portion of bacteria from BAP or a half-portion of bacteria from the *C. diff*-positive CCMA-TAL_plate1 and inoculate bacteria into a 1.5mL microcentrifuge tube containing 750µL PBS.
NOTE: If proceeding immediately to DNA Extraction, inoculate bacteria into 500µL PBS and omit steps 5.4-6.2.

5.3 Use a new 10µL loop to collect all bacterial colonies from BAP and inoculate into cryovials containing 1mL Nutrient Broth + 20% glycerol for long term storage at -80°C.

5.4 Centrifuge inoculated microcentrifuge tubes at 6,000×*g* for 10 min.

5.5 Remove and discard supernatant without disturbing the pellet.

5.6 Store bacterial pellets at -20°C.

6 DNA Extraction on KingFisher Apex Instrument (based on Applied Biosystems “MagMax DNA Multi-Sample Ultra 2.0 Kit User Guide” with modifications)

6.1 Starting material: bacterial pellets from step 5.6 or bacterial suspensions from step 5.2.

6.2 Resuspend pellets in 400µL PBS.

6.3 Prepare 80% Ethanol (2mL per sample, plus overage).

6.4 Prepare a 50mg/mL lysozyme (ThermoFisher 89833) solution by resuspending lysozyme powder in 35% glycerol solution (40µL per sample, plus overage).

6.5 Prepare 1:10 Binding Bead Mix using Lysis/Binding solution (ThermoFisher A36581) and DNA Binding Beads (ThermoFisher A36579) (440µL per sample, plus overage).

6.6 Prepare 96-well extraction plates by adding solution listed below into all corresponding wells that will contain a sample:

Wash Plate 1 - 1000µL Wash Solution 1 (ThermoFisher A36580)
Wash Plate 2 - 1000µL 80% Ethanol
Wash Plate 3 - 1000µL 80% Ethanol
Elution Plate - 100µL Elution Solution (ThermoFisher A36582)
Tip Comb - place tip comb into deep well plate
Sample Plate - 40µL lysozyme solution

6.7 Finish preparing Sample Plate by adding 400µL sample into appropriate wells according to sample template.

6.8 Start pre-saved "MM_Ultra2_Bac_Yeast_Blood" program on KingFisher Apex instrument. Program provided by ThermoFisher.

6.9 Load plates when prompted.

6.10 When instructed (about 20 min into the run), remove sample plate and add 40µL Enhancer Solution (ThermoFisher A36583) to the bottom of each well containing a sample, and 40µL Proteinase K (ThermoFisher A36578) to the top of each well containing a sample.

6.11 Place plate back into instrument and press start.

6.12 When instructed (about 20 min after step 6.11), remove sample plate and add 440µL Binding Bead Mix to each well containing a sample.

6.13 Note: Before adding, invert the Binding Bead Solution to mix. Add slowly.

6.14 Place plate back into instrument and press start.

6.15 While instrument is running, label 1.5mL microcentrifuge tubes with sample IDs.

6.16 When run is finished (about 30 min after step 6.14), remove plate from instrument and transfer eluted DNA into the pre-labelled 1.5mL microcentrifuge tubes for storage (4°C short term, -20°C long term).

6.17 Perform DNA quantification for each sample using Qubit with the dsDNA broad range assay kit (ThermoFisher Q32853).

Library Preparation

7 **Illumina DNA Prep (M) Tagmentation Kit - 96 Samples (Illumina 20060059) NOTE: Protocol based on "Illumina DNA Prep Reference Guide" with modifications**

8 **Tagment Genomic DNA**

8.1 Prepare the "TAG" program on the thermal cycler:

Preheat lid to 100°C
Set the reaction volume to 40µL
55°C for 15 min
Hold at 10°C

8.2 Prepare "Tagmentation Master Mix" - Calculate 10% overage.

	Reagent	Storage	Instructions	Amount per Sample
	BLT	2 to 8°C	Bring to RT, vortex	5µL
	TB1	-25 to -15°C	Bring to RT, vortex	5µL

Note: RT = room temperature

8.3 Remove EPM reagent from freezer (-25 to -15°C) and thaw on ice.

8.4 Remove index plate (Illumina DNA/RNA UD Indexes Set A Tagmentation - 96 Samples; 20091654) from freezer (-25 to -15°C) and thaw at room temperature.

NOTE: Any of the Illumina DNA/RNA UD index plates (Set A, B, C, or D) can be used.

8.5 Place DNA samples on ice to keep cold.

8.6 Add 1-30µL DNA to each well of a 96-well PCR plate so that the total amount is 100-500ng.

8.7 If DNA volume in step 8.6 is < 30µL, add nuclease-free water to bring the total volume of each well to 30µL.

8.8 Vortex prepared "Tagmentation Master Mix" thoroughly.

8.9 Divide "Tagmentation Master Mix" evenly into an 8-tube strip.

8.10 Using a multichannel pipette, add 10µL "Tagmentation Master Mix" to each well of the PCR plate containing a sample.

8.11 Seal plate with 'Microseal B' (Fisher 509010045).

8.12 Place PCR plate on a plate shaker (IKA MS 3) at 1800rpm for 1 min.

8.13 Spin the PCR plate briefly in a centrifuge (~3 sec).

8.14 Place the sample plate in thermal cycler and run the "TAG" program (step 8.1).

9 Post Tagmentation Cleanup

9.1 Prepare the "PTC" program on the thermal cycler:

Preheat lid to 100°C
Set the reaction volume to 50µL
37°C for 15 min
Hold at 10°C

9.2 Remove the sample plate from thermal cycler and spin down briefly (step 8.13).

9.3 Remove 'Microseal B' from sample plate.

9.4 Divide TSB reagent evenly into an 8-tube strip.

9.5 Add 10µL TSB to each well of the sample plate containing a sample.

9.6 Seal plate with a new 'Microseal B'.

9.7 Place plate on plate shaker at 1800 rpm for 1 min.

9.8 Spin down briefly (~3 sec).

9.9 Place PCR plate in thermal cycler and run the "PTC" program (step 9.1).

9.10 Remove sample plate from thermal cycler and spin down briefly (step 8.13).

9.11 Remove 'Microseal B' from sample plate and place plate onto magnetic stand.

9.12 Wait until liquid is clear (3 min).

9.13 Using a multichannel pipette, remove and discard supernatant.
NOTE: do not disturb the beads.

9.14 Wash two times (steps 9.15-9.19):

9.15 Remove sample plate from magnet and add 100µL TWB to each well.

9.16 Seal plate with Optical Adhesive Film (ThermoFisher 4311971) and shake at 1800 rpm for 1 min.

9.17 Spin down briefly (step 8.13).

9.18 Remove seal and place plate onto magnetic stand.

9.19 Remove and discard supernatant.
NOTE: After second wash, do not remove supernatant until step 10.2.

9.20 During downtime in second wash step, prepare "Enhanced PCR Master Mix" - Calculate 10% overage.

9.21

	Reagent	Storage	Instructions	Amount per Sample
	EPM	-25 to -15°C	Thaw on ice, invert to mix, briefly centrifuge	10µL

Reagent	Storage	Instructions	Amount per Sample
Nuclease free H2O	RT	-	10µL

10 **Amplify Tagmented DNA**

10.1 Prepare the "BLT PCR" program on the thermal cycler:
Preheat lid to 100°C
68°C for 3 min
98°C for 3 min
(X) cycles:
 98°C for 45 sec
 62°C for 30 sec
 68°C for 2 min
68°C for 1 min
Hold at 10°C

Recommended number of PCR cycles per DNA input:

Total DNA input (ng)	Number of PCR cycles (x)
1-9	12
10-24	8
25-49	6
50-99	5
100-500*	5

*Recommended input

10.2 Vortex "PCR Master Mix" and briefly spin down.

10.3 Remove sample plate from magnetic stand.

10.4 Immediately add 20µL "PCR Master Mix" to each sample well.

10.5 Seal plate with Optical Adhesive Film and shake at 1800 rpm for 1 min.

10.6 Spin down briefly (step 8.13).

10.7 Add 5µL of appropriate index adapters to each sample.

10.8 Seal plate with Optical Adhesive Film and shake at 1800 rpm for 1 min.

10.9 Spin down briefly (step 8.13).

10.10 Place plate in thermal cycler and run "BLT PCR" program (step 10.1).

10.11 **NOTE:** safe stopping point; sample plate can be stored in the fridge (2°C - 8°C) for up to 3 days.

11 **Clean Up Libraries**

11.1 Prepare Diluted IPB solution.

	Reagent	Storage	Instructions	Amount per Sample
	IPB	RT	Vortex thoroughly before each use	45 µL
	Nuclease- free H2O	RT	-	65 µL

11.2 Prepare 80% Ethanol (400µL per sample, plus overage).

11.3 Remove RSB reagent from freezer (-25°C to -15°C) to thaw and bring to RT. Vortex to mix.

11.4 Remove sample plate from thermal cycler and centrifuge briefly to collect contents at the bottom of the wells.

11.5 Place sample plate on magnetic stand (5 min).

11.6 Transfer 20µL supernatant from each sample well to the corresponding well of a new PCR plate ("midi plate"; Fisher 14-230-244).

11.7 Thoroughly vortex diluted IPB solution and add 110µL to each well containing a sample.

11.8 Seal plate with Optical Adhesive Film and shake at 1800 rpm for 1 min.

11.9 Incubate at RT (5 min).

11.10 Place "midi plate" on magnetic stand (5 min).

11.11 During downtime, thoroughly vortex stock (undiluted) IPB and add 15µL to each well of a new PCR plate ("size selection"; Fisher E0030129504).

11.12 Transfer 125µL supernatant from the "midi plate" to the corresponding well of the "size selection" plate containing the IPB.

11.13 Seal plate with Optical Adhesive Film and shake at 1800 rpm for 1 min.

11.14 Incubate at RT (5 min).

11.15 Place "size selection" plate on magnetic stand (5 min).

11.16 Without disturbing the beads, remove and discard supernatant.

11.17 Wash two times (steps 11.18-11.20).

11.18 While plate is on magnetic stand, add 200µL of 80% ethanol.

11.19 Incubate 30 sec.

11.20 Without disturbing the beads, remove and discard supernatant.

11.21 Use a 10-20µL multichannel pipette to remove residual ethanol.

11.22 Air-dry on magnetic stand (5 min).

11.23 NOTE: Inspect plate after 5 min and add additional time if needed; do not let the beads dry out!

11.24 Remove plate from magnetic stand.

11.25 Add 32µL RSB to beads.

11.26 Seal plate with Optical Adhesive Film and shake at 1800 rpm for 1 min.

11.27 Incubate at RT (2 min).

11.28 Place plate on magnetic stand (2 min).

11.29 Transfer 30µL supernatant to a new 96-well PCR plate ("final DNA libraries").

11.30 Seal plate with Optical Adhesive Film.

11.31 Store at -25°C to -15°C for up to 30 days OR proceed directly to Step 12.

Pool Libraries

12 **Pool Libraries**

12.1 Combine 5µLof each library per column into a 1.5mL microcentrifuge tube.

12.2 Vortex briefly and spin down.

12.3 Quantify each library pool concentration using a dsDNA High Sensitivity Qubit Kit (ThermoFisher Q32854).

12.4 Convert qubit concentrations from ng/µL to nM.

$$\frac{\text{concentration (ng/}\mu\text{L)}}{660\ \times\ fragment\ size\ (bp)} \times 1000000$$

Note: use standard 700bp fragment size for calculation OR as determined by user

12.5 Normalize each pool to an equimolar concentration; dilute with RSB.

12.6 Vortex briefly and spin down.

12.7 Combine 15µL of each library pool into a single 1.5mL microcentrifuge tube. This is the final DNA library pool to be sequenced.

Whole Genome Sequencing (WGS)

13 **Choose WGS instrument based on sample count:**
48-72 samples = NextSeq1000 P1 XLEAP-SBS 300-cycle kit (2×150bp reads)
73-192 samples = NovaSeq X Plus V1, 300-cycke kit (2×150bp reads)

13.1 Perform Tapestation using D1000 screen tape on the final library pool to determine fragment size distribution.

13.2 Spike DNA library pool with 4% PhiX (NextSeq1000).

13.3 Dilute final library pool to final loading concentration;
NextSeq1000 = 850pM or NovaSeq X Plus = 300pM.

13.4 Load libraries and reagents into appropriate instrument.
