# Supplementary material for: Methods for cost-efficient, whole genome sequencing surveillance for enhanced detection of outbreaks in a hospital setting
Source: PLoS One. 2026 Sep 1;21(9):e0355550. doi: 10.1371/journal.pone.0355550 (PMC13533342; doi:10.1371/journal.pone.0355550)
Supplement: Supplementary file 3 [file pone.0355550.s002.docx]

**Supplementary Table 2. Phase I and II sequencing metrics per platform.** The sequencing metrics are provided by the manufacturer and what was determined by the Microbial Genomic Epidemiology Laboratory (MiGEL) during three years of real time genomic surveillance. For MiGEL, the average metrics are provided along with the metrics provided by the manufacturer (Illumina). **^‡^**Note 1, MiGEL-specific metrics were computed and reported as average [standard deviation]

*****Note 2, A standardized service allocation of approximately 350 million reads was used for our samples on the NovaSeq X Plus flowcell, from our sequencing vendor (Commercial Lab 2).

**^#^**Note 3, Expected turnaround time for NovaSeq X Plus (Commercial Lab 2) was 5 business days or less from sample receipt. The manufacturer metrics were obtained from the following websites:

a) <https://www.illumina.com/systems/sequencing-platforms/miseq/specifications.html>,

b) <https://www.illumina.com/systems/sequencing-platforms/nextseq/specifications.html>,

c) <https://www.illumina.com/systems/sequencing-platforms/nextseq-1000-2000/specifications.html>,

d) <https://www.illumina.com/systems/sequencing-platforms/novaseq-x-plus/specifications.html>.

|  | | **Phase I** | | **Phase II** | |
| --- | --- | --- | --- | --- | --- |
|  |  | **MiSeq^a^** | **NextSeq 550^b^** | **NextSeq 1000^c^** | **NovaSeq X Plus^d^** |
|  |  | 2×300 bp (v3) | 2×150 bp (v2.5, mid) | 2×150 bp  (P1 XLEAP-SBS) | 2×150 bp  (X Series 25B) |
| Sample  Count | **MiGEL^‡^** | 32 [1.85] | 65 [16.17] | 70 [15.27] | 84 [18.78] |
| Run Time (hours) | **MiGEL^‡^** | 33 [20.87] | 25 [0.75] | 16 [0.62] | Not provided**^#^** |
|  | **Manufacturer** | 56 | 26 | 17 | 48 |
| Yield (gigabases) | **MiGEL^‡^** | 12 [1.5] | 40 [8.21] | 38 [8.4] | 117 [35.73] |
|  | **Manufacturer** | 13.2-15 | 32.5-39 | 30 | 8,000-10,500 |
| Output  (reads) | **MiGEL^‡^** | 77 [9.77] million | 257 [51.39] million | 244 [13.43] million | 392 [119.09] million***** |
|  | **Manufacturer** | 44-50 million | 260 million | 200 million | 52-70 billion |
